# One-Year Follow-Up Lung Ultrasound of Post-COVID Syndrome—A Pilot Study

**DOI:** 10.3390/diagnostics13010070

**Published:** 2022-12-26

**Authors:** Martin Altersberger, Anna Grafeneder, Yerin Cho, Roland Winkler, Ralf Harun Zwick, Gebhard Mathis, Martin Genger

**Affiliations:** 1 Department of Cardiology, Nephrology and Intensive Care Medicine, State Hospital Steyr, 4400 Steyr, Austria; 2 Rehabilitation Center Hochegg for Cardiovascular and Respiratory Diseases, 2840 Grimmenstein, Austria; 3 Therme Wien Med, 1100 Vienna, Austria; 4 Praxis of Internal Medicine, 6830 Rankweil, Austria

**Keywords:** post-COVID-syndrome, lung ultrasound, COVID-19, LUS

## Abstract

(1) Background: Millions of people worldwide were infected with COVID-19. After the acute phase of the disease, many suffer from prolonged symptoms, the post-COVID syndrome, especially the phenotype with lung residuals. Many open questions regarding lung ultrasound (LUS) have to be answered. One essential question is the means for optimal following-up of patients with post-COVID-19 residuals with LUS; (2) Methods: A retrospective data analysis of patients after acute COVID-19 infection diagnosed with post-COVID syndrome in the state hospital of Steyr and the rehabilitation center of Hochegg was performed. LUS examinations following a 12-zone scanning protocol were performed, and the LUS score quantified comet tail artifacts. A total of 16 patients were evaluated twice with LUS from May 2020 until June 2021. (3) Results: All patients’ reverberation artifacts were reduced over time. The initial LUS score of 17.75 (SD 4.84) points was decreased over the duration of the second rehabilitation to 8,2 (SD 5.94). The difference in the Wilcoxon test was significant (*p* < 0.001); (4) Conclusions: Lung ultrasound was a valuable tool in the follow-up of post-COVID-syndrome with lung residuals in the first wave of COVID-19. A reduction in reverberation artifacts was demonstrated. Further studies about the clinical significance have to follow.

## 1. Introduction

The COVID-19 pandemic is an immense burden on healthcare workers [1]. Millions of people have been infected, there are continuously rising numbers, and new virus variants are still being discovered. Likewise, the follow-up after COVID-19 presents some difficulties. Some patients suffer from ongoing symptoms such as dyspnea after acute COVID-19, even though they have a mild form of the disease [2]. In this pilot study of post-COVID syndrome patients, the usage of lung ultrasound (LUS) in the follow-up through identifying the change of reverberation artifacts over the course of approximately one year is evaluated.

LUS can identify persistent reverberation artifacts after a COVID-19 infection, commonly known as B-lines, though the more accurate terminology through etiology is comet tail artifacts [3,4,5].

### 1.1. Background Lung Ultrasound

#### 1.1.1. Reverberation Artifacts

Artifacts in LUS are generated by air. Reverberation artifacts show a fluid/air mismatch. An overload of fluid in the interstitium leads to vertical artifacts, so-called B-lines in pulmonary edema, or comet tail artifacts in infectious and interstitial diseases [4,5,6]. The evaluation of the pleural line is mandatory to differentiate B-lines from comet tail artifacts. A fragmented irregular pleural line points towards inflammation and the presence of comet tail artifacts. Such an example would be COVID-19 [4,7]. A smooth pleural line with vertical reverberation artifacts can be seen in pulmonary edema. Hence, they are called B-lines (Video S1) [4,5]. Still, there is an active discussion about optimally describing such artifacts, and the current terminology is still insufficient [8].

Comet tail artifacts indicate diffuse alveolar damage and subpleural consolidations, and a reduction in lung sliding can also be observed, indicating inflammatory diseases or diffuse parenchymatous lung diseases (Video S2) [4,9,10]. In the context of critical COVID-19 patients, massive reverberation artifacts were found and described as having a light-beam appearance and seeming like a waterfall [11,12].

#### 1.1.2. Consolidations and Pleural Effusion

Hypoechoic areas have a “tissue-like appearance” or “hepatization” in LUS with small hyperechoic [bronchograms] or hypoechoic (fluid bronchograms) structures within them point toward pneumonia and are described as consolidations and can appear with free fluid [pleural effusion] [7,13,14,15,16,17]. Pleural effusions in COVID-19 are rare and can be seen in bacterial superinfections or other diseases such as heart failure [9,12,18,19]. If the consolidation is present in an entire lung lobe, the borders will be well-defined as a space-consuming entity. In moderate or more significant consolidations, the deeper edges appear irregular and like they were “torn” from the lungs, described as the shred sign [6,20].

#### 1.1.3. COVID-19 LUS, Scoring, Prognosis and Follow-Up

Imaging the lungs is helpful in the follow-up of COVID-19, and LUS can be an add-on tool to chest radiographs and CTs [9,18,21,22]. Significantly, in settings where CTs are not readily available, LUS can assist by means of triage [21,22]. Signs of COVID-19 in lung ultrasound are reverberation artifacts known as comet tail artifacts [4,23,24]. In a setting where lung inflammation is present, these comet tail artifacts can be quantified, and a scoring system can be used to predict outcomes in the acute stage of the disease [21,22,25]. Depending on the scoring system used, different cut-off values are described as markers of poor prognosis [21,25].

**Figure 1:**
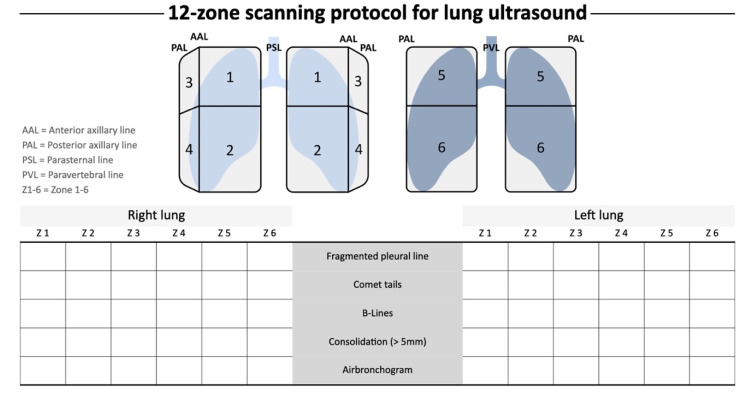
The 12-zone scanning protocol, as suggested by the Austrian Society of Pneumonology and the Austrian Society of Ultrasound in Medicine.

**Figure 2:**
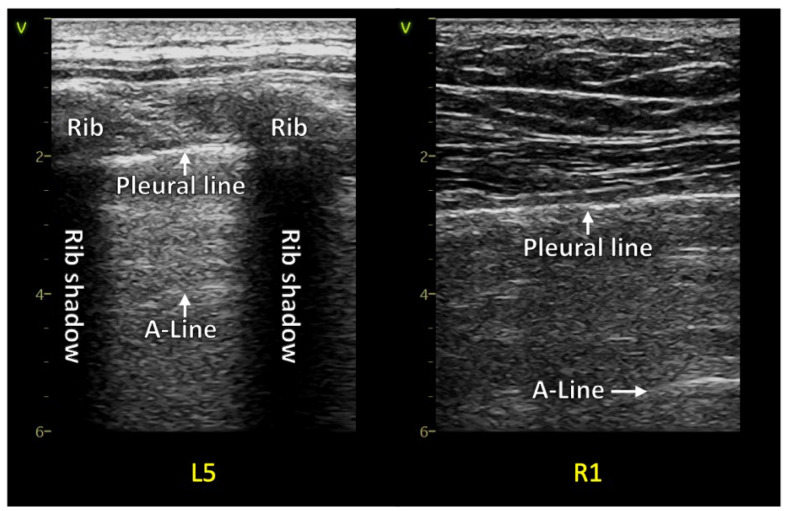
Two normal examples with smooth pleural lines and no reverberation artifacts in post-COVID patients.

**Figure 3:**
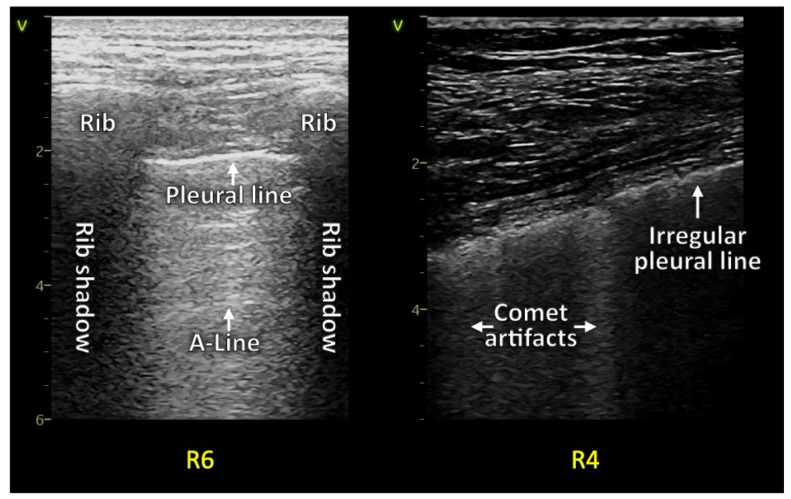
On the left side is a normal image of LUS in post-COVID, and on the right is an irregular pleural line with comet tail artifacts shown.

**Figure 4:**
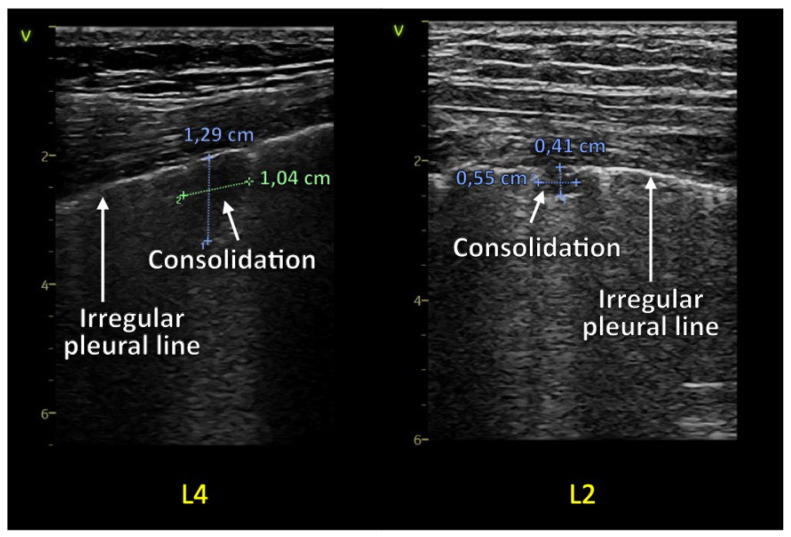
Two areas with small consolidations, including the measurement in the images. On the left side, there is zone four of the left hemithorax (L4), showing an irregular pleural line and a consolidation. On the right side is zone two on the left hemithorax (L2).

The LUS describes a range from 0 to 3 points. 0 points are a typical “physiological” finding of artifacts, meaning normal A-lines and none or few [up to two per intercostal space]. An LUS score of 1 describes a loss of aeration with an irregular pleural line and some comet tails. 2 points is a significant loss of aeration with a markedly irregular pleural line, a reduction in lung sliding, small consolidations located sub-pleural, and many comet tail artifacts. A score of 3 points in the LUS score is given with a large consolidation [25,26,27] (Figure 1, Figure 2, Figure 3 and Figure 4).

### 1.2. Long COVID & Post-COVID Syndrome

There needs to be more data using LUS in post-COVID syndrome patients with lung residuals. The need for rehabilitation is undoubtedly given after critical and severe COVID-19 disease [28,29]. A study by Sonnweber et al. showed that post-COVID-19 patients suffered from persistent symptoms such as dyspnea and impairment in lung function [30]. This study indicated that a follow-up of post-COVID syndrome patients with ongoing dyspnea should include lung imaging with computerized tomography (CT) scans [30].

As the number of symptomatic patients after an acute COVID-19 infection increased, the terms “long COVID” (symptoms after four weeks of acute infection) and “post-COVID syndrome” (symptoms after 12 weeks of acute infection) were introduced and are now used to describe more than 100 different persistent symptoms after acute infection [7,31,32,33]. Pathologic chest CTs were found in 35% of patients 60 to 100 days after initial presentation, and pathological X-rays were present in approximately two-thirds of hospitalized COVID-19 patients [31,34].

## 2. Materials and Methods

### 2.1. 12-Zone LUS Scanning Protocol for Follow-Up

A 12-zone scanning protocol for the follow-up was implemented, describing the artifacts and consolidations with the suggested terminology provided in a review endorsed by the Austrian Society of Ultrasound. The Austrian Society of Pulmonology initially described this in a WFUMB position paper, which deals explicitly with the etiologic differentiation of LUS artifacts [4,7]. LUS was implemented during the initial visitation at the treatment center and in the follow-up as a part of the standard ultrasound evaluation in post-COVID syndrome care in combination with a routine echocardiographic exam [7]. 

The 12-zone scanning protocol was always used and performed with the same standard evaluation. Trained personnel in LUS with at least four years of experience scanned the patients at the respective centers. Additionally, to compare findings over time, the LUS score was calculated after the exam with a second look at the designated workstation, measuring the size of found consolidations (GE healthcare EchoPAC) [26,27]. 

The exams were reviewed in echo laboratories certified by the Austrian Society of Cardiology, division of echocardiography. The protocol started in zone one in supine-positioned patients. The anterior and cranial zone of the respective hemothorax was scanned first (Figure 1). Initially, the anterior and lateral right hemithorax was scanned, saving loops of a longitudinal orientation of the transducer [the marker of the transducer pointing cranially] and a transverse orientation [the marker pointing towards the right side of the patient]. There was a particular focus on scanning all the intercostal spaces in two orientations. In the case of pathological findings, loops of reverberation artifacts and their distribution and consolidations were saved as loops. The examiner was always located on the right side of the patient. The midline of the thorax was the border between zone one and two, and the anterior axillary line marked the border between the anterior and the lateral zones (Figure 1). For the posterior zones, patients were moved to an upright position. Zones five and six on the posterior side were examined and documented in the same way as the anterior and lateral zones.

### 2.2. Image Acquisition

A retrospective data analysis of patients in care after acute COVID-19 infection diagnosed with “long COVID” or “post-COVID syndrome” in the state hospital of Steyr and the rehabilitation center of Hochegg was performed. Lung ultrasound examinations following a 12-zone scanning protocol as priorly described were performed, and the LUS score was quantified utilizing reverberation artifacts (comet tails) and consolidations. Examples are seen in figures two with normal LUS anatomy and three and four with pathologic examples. A specific lung preset with a low mechanical index, single-focal point modality, and without harmonic imaging or other cosmetic filters was set with the focal zone placed at the area of the pleural line [7,35]. The frequency of the LUS preset with a linear transducer was set at 8 to 10 megahertz. The abdominal transducer was set at 4 to 6 megahertz. The VIVID S70 and VIVID T8 ultrasound machines from GE healthcare were used to acquire images. 

A linear transducer with a dedicated LUS preset was used in a standard exam. Depth settings were adapted within a range of 4 to 8 cm. A convex transducer with a depth setting of 6 to 15 cm was selected in cases of larger patients and for quantifying pleural effusions or consolidations [7,36]. For larger patients, a cardiac transducer would have been applicable as well. The gain settings were adjusted to optimally visualize pathologic findings (Figure 3 and Figure 4) [8].

### 2.3. Study Population

A total of 16 patients were included for statistical evaluation. The patients were evaluated twice with LUS from May until June 2020 and in a follow-up visitation around 2021. The mean age was 60 years, ranging from 35–83 years. Out of all the patients, 13 were male, and three were female. Arterial hypertension was present in 11 patients, and diabetes mellitus type II in 3 (Table 1).

Percentages, standard deviation, and mean values were described for descriptive statistics. The Wilcoxon test was applied for the hypothesis that there is no difference in LUS-imaging in two coherent time points in post-COVID syndrome and the alternative hypothesis that there is a difference. As it was paired and not normally distributed in the follow-up rehabilitation, the Wilcoxon test was chosen over the t-test. 

Table 2 displays the minimum and maximum numbers of the individual LUS scores with the mean value of all 16 patients. Table 3 describes the details of evaluating the 12 zones with all the values added for the 16 patients.

For all the statistical analysis, the statistical software SPSS was used. The statistical software SPSS (Version 28, IBM, New York, NY, USA) was used for data analysis. Informed consent for all published material was obtained.

## 3. Results

A total of 16 post-COVID patients were evaluated on two occasions. The overall patient characteristics are displayed in Table 1.

The exams took place from the first time in May until June 2020. The follow-up visitation was carried out around 2021. Twelve [75%] of the patients had well-controlled hypertension as a prior known disease. Eight [50%] had LV hypertrophy, three had diabetes mellitus type two, and three had atrial fibrillation [both 18.75%]. None had known lung or other than previously mentioned diseases, and none were smokers. Of the 16 patients, two were female, and 14 were male [Table 1]. The mean BMI was 28.13 ranging from approximately 22 to 38 [Table 1].

The first scan was performed 1–5 months after severe or critical COVID-19 pneumonia. The second evaluation was conducted 5–13 months later.

In our cohort, in all patients, comet tail artifacts and consolidations were quantified by the LUS score. We described a change in pathological findings over 6–13 months. Both times, a standard lung ultrasound approach with a 12-zone scanning protocol was chosen (Figure 1) [7]. All patients did show some mild remaining pathological findings. Two of them had a persistent LUS score above 20 points. The median at the initial scan of 18 was reduced to 8 points in the follow-up. Most consolidations vanished over time, as well. One small anterior consolidation did persist (anterior hemithorax on the right side—zone one). The detailed occurrence of consolidations is displayed in Table 4. All consolidations were small.

Interestingly, in the follow-up scan, one consolidation was found in an area (left posterior hemithorax—zone five) where it was not seen in the initial scan. Due to the anatomical location of the scapula, this small consolidation might have been missed in the initial scan. Pleural effusions were not seen in the evaluated patients after COVID-19.

There were significantly more artifacts and consolidations in the initial evaluation compared to the second evaluation. By means of consolidation, most were found in the posterior basal regions (left and right number six, Table 4). 

In Table 4, all consolidations found in LUS at the first and second visitation are displayed in the specific region of scanning in which they appeared.

Table 2 shows the minimum and maximum LUS scores at the first and second evaluations. The mean values in both instances are visualized as well. At the initial assessment, the minimum score of all zones added was 10. The maximum score was 27. In the follow-up, the minimum LUS score was 2, and the maximum score was 23. 

**Figure 5:**
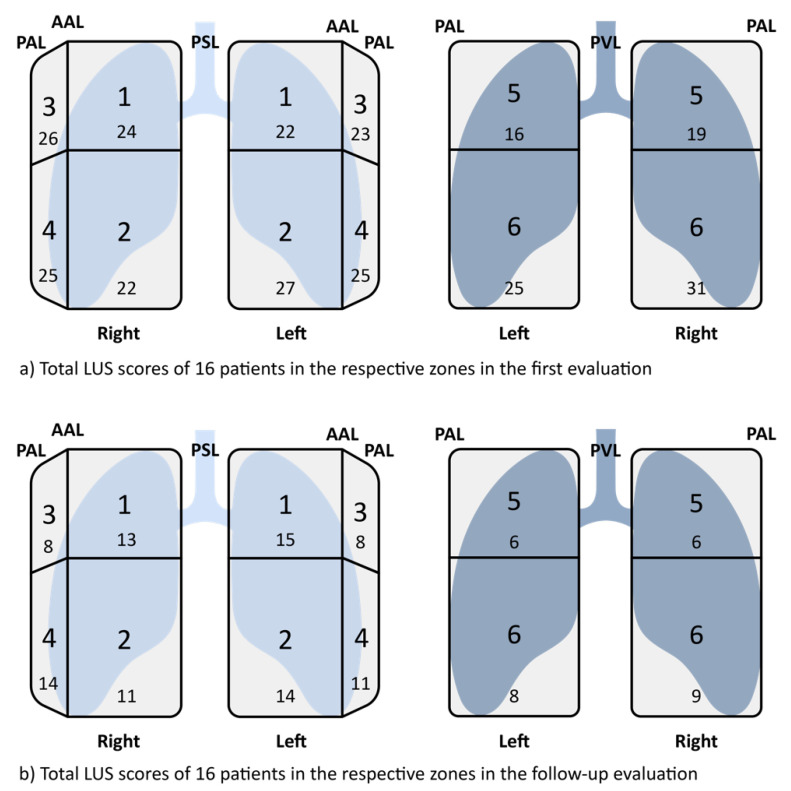
(**a**) Total LUS scores of 16 patients in the respective zones in the first evaluation/ (**b**) Total LUS scores of 16 patients in the respective zones in the follow-up evaluation.

Table 3 and Figure 5 show the detailed analysis of the added number of individual patients showing that at the time of the first evaluation, the LUS score was higher compared to the second evaluation, with the highest score at zone six on the right side and zone two at the left hemithorax. In the second evaluation, interestingly, the most points were found at both hemithoraces in anterior regions (zone one and two on the left side and zone one on the right side as well as zone four on the right side).

The reverberation artifacts at the first evaluation displayed in all 16 patients a mean of approximately 17.75 (SD 4.8). In the follow-up, it decreased to 8.19 (SD 5.9) (graphic 2). The overall mean LUS scores were reduced by 46%. 

The Wilcoxon test in the evaluation of the first and the second LUS did show a significant reduction in comet tail artifacts utilizing the LUS score (*p* ≤ 0.001). Interestingly, none of the included patients showed an entirely normal LUS imaging of the lung. Some reverberation artifacts were persistent in all patients.

## 4. Discussion

Patients with a post-COVID syndrome can face ongoing symptoms over a long period, including dyspnea [6,7,8,26]. In a study by Sonnweber et al., it was concluded that serial testing should be considered in patients with persistent symptoms, including serial lung function testing, echocardiography, and CT imaging [30]. LUS has been a more widely used tool in recent years in pulmonary imaging to identify pathological changes in COVID-19 pneumonia in the acute setting [3,5,9,20,36,37,38]. LUS can function as one of four specified parameters to provide information on hospital admission in acute COVID-19 disease alongside cardiovascular disease, day of illness, and leucocyte count [39]. 

As there are recommendations for LUS during the acute setting, there are also recommendations for follow-up [3,7].

This pilot study used a 12-zone scanning protocol in LUS to describe comet tail artifacts and consolidations after acute COVID-19 disease. To our knowledge, this is the first study to follow-up patients after a hospital stay or critical COVID-19 from the first COVID-19 wave to demonstrate existing reverberation artifacts as sequelae of lung disease. A recent study did show persisting reverberation artifacts after six months of acute COVID-19 disease and compared it with CT scans of the lungs. It is demonstrated that persistent changes in lung imaging (CT & LUS) are seen after six months, but the clinical relevance is unclear. The authors suggest that LUS might be a screening tool to rule out relevant lung diseases after COVID-19, such as pulmonary fibrosis [40,41,42]. In an ambulatory setting or rehabilitation, LUS can be a low-cost, easy-to-use, widely available, and radiation-sparing alternative to serial chest radiographs or CT scans for tracking improvement [3,7,40]. 

By means of prognosis, a score of 26 is considered a cut-off if a patient is scanned in the emergency department to have a 90% specificity of a lethal outcome during this admission. A score of 25 is associated with being admitted to the ICU, with a specificity of 90% in admitted patients due to COVID-19 [25]. In a study by Lichter et al., a LUS score of 18 or more seemed to be a cut-off for the follow-up in terms of mortality and requirement for ventilatory support [22]. 

The previously mentioned scores are in the case of a 12-zone LUS scanning protocol. As there are several protocols in use and some show 14 zones per each hemithorax, different values have to be known for the cut-off by means of prognosis [21,35]. In a study by Skopljanac et al., it was described that the LUS score was associated with oxygen demand at admission. Similar to a 12-zone scanning protocol, high LUS score values predicted more intensive respiratory care. A score of 29 was a cut-off for high-flow oxygen therapy and 30 for mechanical ventilation [21]. In our cohort, high scores after severe COVID-19 pneumonia were found and significantly reduced in the follow-up.

## 5. Limitations

Some limitations have to be addressed. First of all, it has to be pointed out that a very heterogeneous group of patients with quite varying time intervals in between scans were evaluated. The initial follow-up time differed by four months. The follow-up exam was conducted with a variation in time by eight months. The time intervals were scheduled in a real-life clinical scenario during the pandemic with limited resources and aimed to find the optimal timing for all individual patients. A third rehabilitation in a follow-up setting was planned. Still, practically all participants were lost to follow-up as the clinical status improved, and the need for another rehabilitation in the next year (2022) was not applicable.

Previously mentioned differences in settings may cause differences in artifact interpretation. That is a pitfall we encourage not to be overseen in daily clinical practice [8].

Another limitation is that this is a study where the data was evaluated retrospectively. The sample size of 16 patients is small, with a relatively broad range of ages. Besides, only two included patients were female, and the other fourteen were male. A healthy control group is missing, as this was in an ambulatory and rehabilitative setting. 

We encourage all colleagues fighting against COVID-19 to evaluate patients in rehabilitation or in an ambulatory setting with lung ultrasound. 

## 6. Conclusions

LUS might be an easily available and comparatively cheap tool without side effects that can be used in an ambulatory or hospital setting to follow up on COVID-19 patients that are hospitalized or in intensive care units after severe or critical COVID-19. Possible early detection of long-term complications such as pulmonary fibrosis might be in the scope of a thorough LUS exam using a standardized protocol [7,40,43,44]. In this pilot study, it was shown that LUS could add information regarding imaging. Whether the changes in imaging correlate to outcomes and clinical improvement is a topic for further evaluation.

## Figures and Tables

**Table 1:** Patient characteristics of post-COVID syndrome patients.

Age	Range: 35–83
	Mean: 61
Gender	Male: 87.5%
	Female: 12.5%
BMI	Range: 22–38
	Mean: 28.13

0: The mentioned disease is not present. 1: The mentioned disease is present.

**Table 2:** LUS score at the first and second evaluation.

LUS Score	N	Minimum	Maximum	Mean	Standard Deviation
1st evaluation	16	10.00	27.00	17.7500	4.83735
2nd evaluation	16	2.00	23.00	8.1875	5.93542

**Table 3:** LUS score of the 12 zones (zone 1 until 6—right (R) hemithorax, zone one until six left (L) hemithorax) during the first and second evaluation of 16 patients. The mean, the median, and the total values are displayed.

**Zone**	**R1**		**R2**		**R3**		**R4**		**R5**		**R6**	
Evaluation	1st	2nd	1st	2nd	1st	2nd	1st	2nd	1st	2nd	1st	2nd
N	16	16	16	16	16	16	16	16	16	16	16	16
Mean	1.5000	0.8125	1.3750	0.6875	1.6250	0.5000	1.5625	0.8750	1.1875	0.3750	1.9375	0.5625
Median	1.5000	1.0000	1.0000	1.0000	2.0000	0.0000	1.0000	1.0000	1.0000	0.0000	2.0000	0.5000
Total	24.00	13.00	22.00	11.00	26.00	8.00	25.00	14.00	19.00	6.00	31.00	9.00
**Zone**	**L1**		**L2**		**L3**		**L4**		**L5**		**L6**	
Evaluation	1st	2nd	1st	2nd	1st	2nd	1st	2nd	1st	2nd	1st	2nd
N	16	16	16	16	16	16	16	16	16	16	16	16
Mean	1.3750	0.9375	1.6875	0.8750	1.4375	0.5000	1.5625	0.6875	1.0000	0.3750	1.5625	0.5000
Median	1.0000	1.0000	2.0000	1.0000	1.5000	0.0000	2.0000	1.0000	1.0000	0.0000	1.0000	0.0000
Total	22.00	15.00	27.00	14.00	23.00	8.00	25.00	11.00	16.00	6.00	25.00	8.00

**Table 4:** Consolidations found in LUS at the first and second visitation, R [right hemithorax], L [left hemithorax].

Zone	N	Consolidations	
		1st Evaluation	2nd Evaluation
1	R	1	1
	L	0	0
2	R	0	0
	L	1	0
3	R	1	0
	L	0	0
4	R	2	0
	L	1	0
5	R	0	0
	L	0	1
6	R	4	0
	L	2	0

## Data Availability

Not applicable.

## Supplementary Materials

List of videos https://www.mdpi.com/article/10.3390/diagnostics13010070/s1: Video S1: A normal LUS (scanning one anterior area on the right side (zone r2) in a 12-zone scanning protocol) on the left side of the video with several B-lines in pulmonary congestion on the right side of the video in a heart failure patient. Video S2: A normal LUS (scanning one anterior area on the right side (zone r2) in a 12-zone scanning protocol) on the left side of the video and comet tails adjacent to a small area of consolidation in critical COVID-19 on the right side of the video in a patient without other known diseases. The A-lines have vanished and only comet tails are seen. Video S3: LUS changes in post-COVID syndrome from initial visitation to the second visitation six months later. Initially, there are plenty of comet tail artifacts. After six months, the pleural line looks smooth, and there are one or two reverberation artifacts seen. Both exams show the area of the right anterior hemothorax (zone one on the right hemithorax). Video S4: LUS changes in post-COVID syndrome from initial visitation to the second visitation 11 months later. Initially, there are plenty of comet tail artifacts. After six months, the pleural line looks smooth, and there are one or two reverberation artifacts seen. Both exams show the area of the right anterior hemothorax (zone one on the right hemithorax

## Abbreviations

LUS	Lung ultrasound
CT	Computerized tomography
LV	Left ventricle
COPD	Chronic obstructive lung disease
